# “Defining the Independence of the Liver Circadian Clock” & “BMAL1-Driven Tissue Clocks Respond Independently to Light to Maintain Homeostasis”

**DOI:** 10.3389/fnins.2020.00107

**Published:** 2020-02-25

**Authors:** Mirja Rotinen

**Affiliations:** Department of Health Sciences, Public University of Navarre, Pamplona, Spain

**Keywords:** circadian, peripheral clocks, transcription factor, light-dark cycles, autonomous

## Introduction

Metabolism and the circadian rhythms constitute an inseparable couple. Our 24-h internal clock dictates our sleep-wake patterns, which in turn determines what we eat and when, with a direct impact on our metabolic cycles. These rhythms are nominally self-sustained but can be adjusted to the environment by stimuli, such as light or food, called Zeitgebers (the German word for “time-givers”) (Aschoff, 1965). Like the principal conductor leading a symphonic orchestra, a tiny pack of suprachiasmatic nucleus (SCN) neurons in the brain residing directly above the optic chiasm work as a master coordinator of the rest of the circadian clocks of the body. Changes in light are transmitted directly from the retina to the SCN, which actively synchronize the geophysical and environmental cycle with its own clock before entraining other organs (Schibler and Sassone-Corsi, 2002). Although the molecular mechanisms regulating the brain central clock have been studied extensively, the degree of contribution of individual organs to “timekeeping” is still unclear.

**Figure 1:**
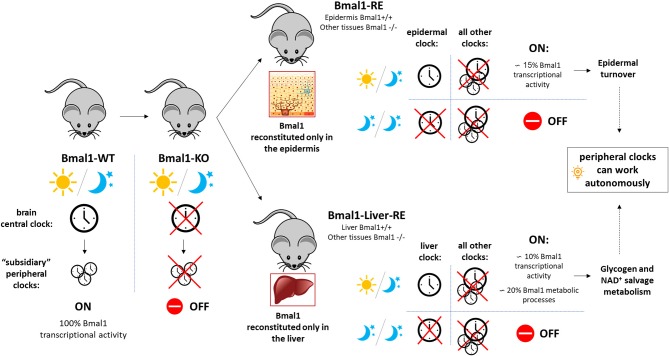
Schematic representation of the main findings from the dual publication (Koronowski et al., 2019; Welz et al., 2019) commented. The mouse model reconstituting Bmal1 expression exclusively in the epidermis (Bmal1-RE mouse) or the liver (Liver-RE mouse) was generated from a conventional full-body Bmal1 knockout mouse (Bmal1-KO). In mammals, the brain central clock works as a master circadian coordinator of the rest of circadian clocks in the body. The autonomous responses in Bmal1 reconstituted organs (epidermis or liver) ensured basic homeostasis of the organs (e.g., epidermal turnover, glycogen metabolism) and were only dependent on inputs of light and darkness. WT, wild type.

The transcription factor BMAL1 (ARNTL) has been shown to be essential for rhythmic gene expression in the mammalian circadian timing system (Haque et al., 2019). BMAL1 forms a heterodimer with CLOCK, another core circadian transcription factor, to drive the expression of the *Period* and *Cryptochrome* genes, by direct binding to E-box regulatory elements (Takahashi, 2017). In a classical transcriptional feedback loop, PER and CRY transcription factors form a complex that translocates to the nucleus to inhibit BMAL1-CLOCK mediated gene expression (Eckel-Mahan and Sassone-Corsi, 2013), ensuring proper functioning of the 24-h molecular oscillator. Bmal knockout mice, in addition to loss of circadian rhythms, show characteristics of premature aging and a number of phenotypes including defective glucose homeostasis, calcification of joints and corneal degeneration (Kondratov et al., 2006). In back-to-back publications in Cell (Koronowski et al., 2019; Welz et al., 2019), the authors go one step further in transgenic engineering and generate a mouse model that reconstitutes Bmal1 expression in one particular organ, such as the epidermis (Bmal1-RE mouse) or the liver (Liver-RE mouse), with Bmal1 being completely absent in any other parts of the mouse body (Figure 1). This provides them with a valuable tool to dissect the unique contribution of peripheral tissues and organs to the master circadian mechanism, as well as to assess their degree of autonomy.

## Liver Circadian Clock Sets Its Own Pace

One of the main discoveries in these studies takes place at the hepatic level (Koronowski et al., 2019). In the liver, many rate-limiting enzymes of key metabolic outputs (including detoxification, carbohydrate, lipid, and amino acid metabolism) are under direct circadian control. In contrast to the master clock, for which light is the dominant Zeitgeber, the liver clock can be efficiently entrained by feeding-fasting rhythms to the point of being fully uncoupled from SCN rhythms (Damiola et al., 2000; Stokkan et al., 2001; Reinke and Asher, 2016). Transcriptomics and metabolomics experiments were conducted in the liver-RE mice mouse model, which exclusively expresses Bmal1 in the liver and compared to wild type animals. These studies revealed a series of metabolic pathways and metabolites that are able to oscillate autonomously from all other clocks, and constitute around 20% of the hepatic rhythms, including essential processes such as glycogen turnover and the NAD^+^ salvage pathway. Importantly, this autonomous response of the liver clock was independent from food, being only disrupted by a prolonged exposure to complete darkness.

## Postulating a Bifid Model for Synchronization of Peripheral Clocks

Some of these findings can be extrapolated to other peripheral tissues, including non-metabolically active organs, as demonstrated in the companion publication on the epidermis (Welz et al., 2019). The skin is the largest organ in the body and a huge sensory light receptor. Bmal1 deficient mouse models have shown premature aging: hair graying, loss of subcutaneous fat layer and delayed tissue healing (Kondratov et al., 2006). Thus, elucidating circadian oscillations in the epidermis could lead to a better understanding of the underlying mechanisms of skin regeneration and optimization of wound healing therapy.

Gene expression profiling in the epidermis from RE mice (with reconstituted Bmal1 expression in the epidermis) revealed a set of core genes which ensure basic homeostasis of the skin independently of Bmal1 expression in other tissues. Reconstituted epidermis accounted for approximately 15% of Bmal1 transcriptional activity (Figure 1). These results were very close to the ones obtained in Liver-RE mice (~10% oscillating transcripts). Gene ontology analysis of the epidermal signature showed enrichment of cell cycle, circadian rhythm, DNA repair and metabolic processes. This core clock machinery appeared to be only dependent on cyclic changes in light to work in a proper manner. This implies that in the absence of light, the signals coming from other organs are essential to maintain the classic 24-h physiological rhythm, with most processes working on a memory mechanism. The authors, thus, postulate that there are two branches that differentially regulate peripheral clock synchronization: an autonomous and immediate response by the organ to the light, and a non-autonomous memory branch that allows the organ to keep working in prolonged darkness.

## Conclusions and Perspectives

Taken together, these studies demonstrate that peripheral tissues and organs can detect changes in environmental light and are capable of maintaining some basic functions, independently from our brain clock (Figure 1). The liver can autonomously ensure glucose homeostasis even if there is a glitch in the feedback system to the central clock (SCN). This could be of critical importance in environmentally challenging conditions to the organism. Our body needs to find a fine balance between diving into adjust its clocks in response to environmental stimuli and opposing change; otherwise we would live in a constant jet lag state. These studies provide evidence of the existence of at least two pathways regulating peripheral circadian clocks to reach that equilibrium. The first is an “immediate and autonomous” response that allows organs to adjust to changes in light, without any input from other circadian clocks. The latter works as a “fail safe copy” of past light regime that guarantees a certain degree of resistance and robustness to environmental changes, which is sustained by signals coming from other organs. Future studies will need to examine how the autonomous circadian clocks become deregulated in pathological contexts (obesity, metabolic disease, diabetes…). Not uncommon these days, the extensive and untimely use of bright screens and electronic devices, is leading to improper exposure to artificial light at the “wrong time.” To what extent this exposure may entail the disruption of the peripheral clocks, predisposing individuals to premature aging or cancer as a consequence of “light misuse” needs to be further addressed.

## Author Contributions

MR conceptualized, wrote, reviewed and edited the manuscript.

### Conflict of Interest

The author declares that the research was conducted in the absence of any commercial or financial relationships that could be construed as a potential conflict of interest.
